# Cross-sectional interactions between quality of the physical and social environment and self-reported physical activity in adults living in income-deprived communities

**DOI:** 10.1371/journal.pone.0188962

**Published:** 2017-12-14

**Authors:** Alexia D. M. Sawyer, Russell Jones, Marcella Ucci, Lee Smith, Ade Kearns, Abi Fisher

**Affiliations:** 1 Department of Behavioural Science and Health, University College London, London, United Kingdom; 2 Glasgow Centre for Population Health, Third Floor, Olympia Building, Bridgeton Cross, Glasgow, United Kingdom; 3 UCL Institute for Environmental Design and Engineering, The Bartlett Faculty of the Built Environment, University College London, Central House, London, United Kingdom; 4 The Cambridge Centre for Sport and Exercise Sciences, Anglia Ruskin University, Cambridge, United Kingdom; 5 Urban Studies, University of Glasgow, Glasgow, United Kingdom; Cinvestav-Merida, MEXICO

## Abstract

**Background:**

Understanding the environmental determinants of physical activity in populations at high risk of inactivity could contribute to the development of effective interventions. Socioecological models of activity propose that environmental factors have independent and interactive effects of physical activity but there is a lack of research into interactive effects.

**Objectives:**

This study aimed to explore independent and interactive effects of social and physical environmental factors on self-reported physical activity in income-deprived communities.

**Methods:**

Participants were 5,923 adults in Glasgow, United Kingdom. Features of the social environment were self-reported. Quality of the physical environment was objectively-measured. Neighbourhood walking and participation in moderate physical activity [MPA] on ≥5 days/week was self-reported. Multilevel multivariate logistic regression models tested independent and interactive effects of environmental factors on activity.

**Results:**

‘Social support’ (walking: OR:1.22,95%CI = 1.06–1.41,p<0.01; MPA: OR:0.79,95%CI = 0.67–0.94,p<0.01), ‘social interaction’ (walking: OR:1.25,95%CI = 1.10–1.42,p<0.01; MPA: OR:6.16,95%CI = 5.14–7.37,p<0.001) and ‘cohesion and safety’ (walking: OR:1.78,95%CI = 1.56–2.03,p<0.001; MPA: OR:1.93,95%CI = 1.65–2.27,p<0.001), but not ‘trust and empowerment’, had independent effects on physical activity. ‘Aesthetics of built form’ (OR:1.47,95%CI = 1.22–1.77,p<0.001) and ‘aesthetics and maintenance of open space’ (OR:1.32, 95%CI = 1.13–1.54,p<0.01) were related to walking. ‘Physical disorder’ (OR:1.63,95%CI = 1.31–2.03,p<0.001) had an independent effect on MPA. Interactive effects of social and physical factors on walking and MPA were revealed.

**Conclusions:**

Findings suggest that intervening to create activity-supportive environments in deprived communities may be most effective when simultaneously targeting the social and physical neighbourhood environment.

## Introduction

It is well-established that sufficient levels of physical activity aid in the reduction of chronic disease, and mortality [1–4]. The United Kingdom (UK) government currently recommends that adults (19–64 years old) are active daily and should accumulate either ≥150 minutes of moderate activity (MPA; e.g. cycling), 75 minutes of vigorous activity (VPA; e.g. running), or a combination, over the week [5]. Additionally, walking 10,000 steps per day is recommended to obtain health benefits [6,7].

However, adults in the UK have low levels of physical activity, even when compared with similar European countries [8,9]. For example, accelerometer data from the Health Survey for England 2008 showed that only 6% of males and 4% of females met national guidelines [10]. Levels of inactivity (i.e. <30 minutes/week of MPA) are particularly high in deprived groups: self-reported data from 163,099 adults in England revealed that levels of inactivity were almost 10% higher in local authorities with the highest levels of socio-economic deprivation compared with the lowest [9]. Even small increases in MPA may be beneficial for inactive groups. A European cohort study including 334,161 adults estimated that moving individuals from inactivity to moderate activity (equivalent to a daily 20-minute walk) produced a 7.35% reduction in all-cause mortality [3].

Understanding the determinants of physical activity, particularly in populations at higher risk of inactivity, could contribute to the development of interventions to increase physical activity. Socioecological approaches to physical activity posit that individual characteristics, the social environment, the physical environment and policies are all key determinants of activity, which are interrelated and embedded in a complex system [11–13]. In addition to having simultaneous independent effects, it is postulated that these influences can also have interactive effects. Growing evidence suggests independent effects of the objectively-measured and perceived aspects of the neighbourhood physical and social environment on physical activity (e.g. [14–17]). However, as Gubbels et al. highlight, while there is a move towards the integration of influences through the use of multivariate models, there is still very limited research examining the *interaction* of factors [18].

Addressing this gap in the literature is an important next step for physical activity research. Firstly, accounting for potential variance arising from hitherto unmeasured interactive effects could help to explain inconsistent results in the literature examining neighbourhood effects. For example, inconsistent effects of crime and aesthetics on physical activity could arise from interactive effects of other aspects of the social and physical environment suppressing or heightening measured effects within certain environmental conditions [19–24]. Secondly, understanding pathways of influence between environmental variables and physical activity could inform development of interventions to create activity-supportive environments in specific contexts, embracing the complexity of environmental influences and ensuring that unmeasured features of the environment do not suppress effects [25]. Examining interactive effects is required to address these points and to test a central tenant of socioecological models of physical activity.

Recent research examining the health effect of micro-scale features of the environment (e.g. disorder, aesthetics, street lighting) in deprived neighbourhoods suggests that disparities in these features between deprived and non-deprived neighbourhoods could contribute to inequalities in health and behaviours [26,27]. Geographic Information Systems (GIS) measures and neighbourhood audits of streetscapes in New York City revealed that more deprived neighbourhoods had poorer aesthetics and safety conditions (e.g. fewer clean streets, fewer landmarked buildings, more felony complaints) than non-deprived neighbourhoods, potentially offsetting macro-scale elements of deprived urban neighbourhoods that ostensibly create walkable environments (e.g. increased land use mix or density) [27]. Results were corroborated by further research in USA demonstrating poorer aesthetics and worse maintenance of the environment (e.g. litter, vandalism) in deprived neighbourhoods with larger populations of racial and ethnic minorities than in higher-income neighbourhoods [26]. Variation in neighbourhood quality between 3 cities (Seattle, San Diego and Baltimore) underscored the necessity of context-specific investigation [26]. Both studies recommended further research examining how features of social and physical environments impact physical activity behaviours, and whether they interact to produce effects, particularly in deprived communities [26,27].

Emerging research in the USA explores interactive associations. Bracy et al. presented few significant interactive effects of perceived safety and perceived physical environment features on objectively-measured moderate-to-vigorous physical activity (MVPA) in a sample from Washington metropolitan areas [28]. However, there was an interaction between perceived safety and walkability: adults who felt unsafe (i.e. reported low levels of perceived safety) and lived in a neighbourhood with low walkability achieved 91.2 fewer minutes of MVPA per week than adults who felt unsafe but lived in a highly-walkable neighbourhood with low walkability. The difference in MVPA between adults living in neighbourhoods with high and low walkability was markedly less (38.8 minutes) among adults reporting high levels of perceived safety, suggesting feelings of safety might have mitigated the effects of walkability. To the best of our knowledge, there is no research examining interactive environmental effects on physical activity in a deprived context in the UK. It is possible that within this context, the social environment and micro-scale features of the physical environment interact in creating an environment which supports physical activity by altering the way in which the space is used or perceived. As such, environmental features could operate synergistically or a feature could modify the effect of the other through mediation or moderation. For example, an individual with higher levels of social interaction in the neighbourhood may be feel more inclined to be active with friends or walk to a neighbour’s house when the local physical environment is attractive and aesthetically-pleasing than when it is not, while the quality of local physical environment has less influence on individuals with fewer social contacts with which to be active. Alternatively, an environment that is clean and orderly might suggest residents abide by social norms, generating feelings of safety and creating an environment in which individuals feel comfortable walking (e.g. [29]).

This study aimed to explore independent and interactive effects of social and physical environmental factors on neighbourhood walking and MPA in adults in income-deprived communities. Focus was on the quality of micro-scale features of the physical environment (disorder, maintenance and aesthetics) and social environment (cohesion, trust, social interaction, social support, participation and safety), furthering previously discussed research exploring the independent effects of these components in deprived settings elsewhere [26,27]. It was hypothesised that higher quality social and physical environments would be independently and interactively associated with increased physical activity.

## Methods

### Population

Participants were adults (aged ≥16 years) taking part in the first wave of data collection of the GoWell programme, a study of the health impact of housing and neighbourhood regeneration in Glasgow, a major city in the UK (http://www.gowellonline.com/). In the UK, the National Health Service classifies individuals over 16 years as (young) adults. Data were collected from 14 inner-city neighbourhoods across the city (**S1 Fig),** comprising 32 sub-areas. All neighbourhoods were classified as income-deprived, with between 25–54% of the neighbourhood population being in receipt of income-related benefits in comparison to the contemporaneous Scottish average of 14% and Glasgow average of 25%. Health and wellbeing profiles (e.g. life expectancy, hospitalization) were broadly similar between study neighbourhoods and other Scottish neighbourhoods with similar levels of deprivation [30]. Neighbourhoods included inner city mass housing estates (mostly comprising high-rise flats), inner suburban garden estates (comprising semi-detached houses and cottage flats) and large peripheral estates (comprising low and medium-rise flats).

Participants were selected by random selection of addresses from the Postal Address File which includes all registered addresses. One adult per household was invited by letter to take part in the GoWell community survey; fieldworkers visited households up to 5 times to seek consent to participation. The survey was conducted at the participant’s home by a trained fieldworker during a 40-minute face-to-face meeting. The response rate to invitations to participate was 50.3%. Compared with national statistics at the time of data collection, in the sample from this wave of data collection in the GoWell study, there were slightly more females (60% of this sample compared with 55% nationally) but comparable levels of individuals identifying as Scottish/British and single-person households. The sample was drawn from neighbourhoods with similar health profiles (e.g. life expectancy and alcohol- and drug-related hospitalisations) to other deprived neighbourhoods in Scotland. Therefore, the sample was deemed to be broadly representative of the target population [30,31]. The GoWell study obtained ethical approval from NHS Scotland B MREC committee (no. 05/MRE10/89). All participants provided informed written consent. Further information on the recruitment process and community survey can be found elsewhere [32,33].

### Measures

Neighbourhood walking was assessed in the GoWell community survey using the item: *‘In a typical week*, *on how many days do you go for a walk around your neighbourhood*?*’* This item did not distinguish between walking for recreational or utilitarian purposes and captured frequency rather than duration of walking periods. Participation in MPA was measured using the item: *‘In a typical week*, *on how many days do you do 30 minutes of moderate physical exercise such as brisk walking*, *cleaning the house–it doesn’t have to be 30 minutes all at once*?’. Responses were collapsed into two binary variables using 5 days as a cut-off (walking/participating in MPA on at least 5 days/week; equivalent to >150 minutes MPA/week), in order to assess whether participants were meeting national recommended guidelines for physical activity [5]. Although participation in vigorous physical activity (VPA) was also measured in the survey, levels of VPA were very low (5% participating in ≥5 days/week). Therefore, only MPA was examined in this study.

The quality of the neighbourhood social environment was self-reported using the GoWell community survey. Items were drawn or adapted from previous surveys including the Home Office Citizenship Survey [34], Scottish Social Attitudes Survey [35], British Social Attitudes Survey [36], Office for National Statistics Measuring Social Capital in the UK [37] and the SHARP Questionnaire [38]. Responses to items assessing diverse aspects of the quality of the social environment (e.g. social support: *‘how many people could you ask to give you advice and support in a crisis*?*’*; community cohesion: *‘to what extent do you agree that this neighbourhood is a place where people from different backgrounds get on well together*?*’*; social interaction: *‘On how many days a week do you speak to your neighbours*?*’*) were scored on 4-6-point Likert scales. The neighbourhood was described to participants as the area within 5–10 minutes’ walk from their home.

A neighbourhood audit collected data on the quality of the physical environment. The audit was conducted by two trained surveyors across 95 randomly-selected postcodes within the GoWell study neighbourhoods. Evaluations pertained to the 100-metre area surrounding the central point of the postcode (comprising the ‘audit site’), encompassing streets, buildings, gardens, paths, fences, outdoor communal areas and public spaces. Items selected from the audit assessed the quality of the physical environment in terms of aesthetics, maintenance and disorder (e.g. ‘*Are buildings marked with graffiti or other signs of vandalism*?’ and ‘*Buildings are clean and fresh looking*’). Items were scored on 4-point Likert scales. Distance to the central point of the nearest audit site was calculated for each participant to permit data linkage (median distance: 151 metres). Audit data were aggregated at the level of the audit site, creating 95 data points.

A principal components analysis was previously conducted on items measuring the social environment and physical environment using this sample. Items measuring the social environment were drawn from the GoWell community survey and items measuring the physical environment were drawn from the neighbourhood audit. Items were retained on a factor if the loading in the pattern matrix was >0.4. Reliability of the scale was deemed satisfactory if ≥0.5, consistent with recommended levels for scales with few items [39]. The analysis obtained 4 factors assessing the quality of the neighbourhood social environment: ‘social support’ (Cronbach’s alpha = 0.9); ‘social interaction’ (Cronbach’s alpha = 0.7); ‘trust and empowerment’ (Cronbach’s alpha = 0.6); ‘cohesion and safety’ (Cronbach’s alpha = 0.5) and 3 factors assessing the quality of the neighbourhood physical environment: ‘aesthetics of built form’ (Cronbach’s alpha = 0.8); ‘cues of physical disorder’ (Cronbach’s alpha = 0.8) and ‘aesthetics and maintenance of open space’ (Cronbach’s alpha = 0.6). Items for each factor are presented in **Table 1**. Each factor was scored from 0.0 to 1.0. These factors were included in analyses and are collectively referred to as environmental factors.

**Table 1:** Items comprising environmental factors.

Environmental factor	Item	Factor loading
‘Social support’	*How many people could you ask to go to the shop for messages (everyday goods) if you are unwell?*	-0.89
	*How many people could you ask to lend you money to see you through the next few days?*	-0.89
	*How many people could you ask to give you advice and support in a crisis?*	-0.89
‘Social interaction’	*How many days a week do you speak to your neighbours?*	0.78
	*How many days a week do you meet up with relatives?*	0.76
	*How many days a week do you meet up with friends?*	0.86
‘Trust and empowerment’	*People who live in this neighbourhood think highly of it*	0.66
	*Someone who lost a purse or wallet around here would be likely to have it returned without anything missing*	0.66
	*On your own*, *or with others*, *you can influence decisions affecting your local area*	0.66
	*Is it likely that someone would intervene if a group of youths were harassing someone in the local area?*	0.62
‘Cohesion and safety’	*To what extent do you agree that this neighbourhood is a place where people from different backgrounds get on well together?*	-0.80
	*To what extent do you feel that you belong to this neighbourhood?*	-0.71
	*How safe would you feel walking alone in this neighbourhood after dark?*	-0.55
‘Aesthetics of built form’	*Buildings are visually interesting (varied in terms of design*, *scale*, *colours*, *textures)*	0.92
	*Buildings are clean and fresh looking*	0.79
	*Area in general is visually interesting (varied in design*, *scale*, *colours*, *textures)*	0.68
‘Physical disorder’	*Buildings show signs of damage or disrepair (not vandalism)*	0.83
	*Private gardens*, *yards and driveways are tidy and well maintained*	-0.66
	*Buildings are marked with graffiti or other signs of vandalism*	0.60
	*Communal areas and public spaces are tidy and well-maintained*	-0.58
	*Area in general is clean and fresh looking*	-0.51
‘Aesthetics and maintenance of open space’	*Communal areas and public spaces are interesting and attractive (i*.*e*. *landscaped)*	0.79
	*Private are gardens are interesting and attractive*	0.66
	*The walls*, *fences or hedges between properties are well maintained*	0.51

Socio-demographics previously found to be associated with environmental factors were included as covariates: sex, tenure, age, citizenship, employment status and neighbourhood income-deprivation (% residents in receipt of income-related benefits). Mobility-limiting illness and vehicle ownership were also included as covariates owing to their possible relationship with physical activity [40,41]. All socio-demographics were self-reported in the GoWell community survey. Neighbourhood income-deprivation was previously calculated at neighbourhood level using the Scottish Index of Multiple Deprivation (SIMD) income deprivation domain [42].

### Statistical analyses

Descriptive statistics characterised the sample by socio-demographics. Chi-square analyses tested for differences in the likelihood of neighbourhood walking or MPA by socio-demographics. A series of multilevel binary logistic regression models were conducted. Model 1 included a single environmental factor, adjusting for covariates and participant sub-area (i.e. postcode). Model 2 included all environmental factors, adjusting for covariates and participant sub-area. Model 3 included main effects and all pairwise interactions between social and physical environment factors, adjusting for covariates and participant sub-area. Using a data-driven approach in line with Aiken and West’s [43] recommendation for exploratory analyses, all pairwise interaction terms were initially entered and insignificant interaction terms were dropped progressively, starting with the least significant term, until only significant terms remained in the model. Post-hoc tests explored significant interactions. Two-level random intercept models accounted for the possibility of clustered responses within sub-areas. Analyses were conducted in Stata 12. Alpha was set at p<0.01, acknowledging the large number of statistical tests.

## Results

Complete data were available for 5,923 participants. Numbers of missing values were low because data were collected face-to-face (<2% of the original sample of 6,008 participants were excluded because of incomplete data). Imputation of missing data was deemed inappropriate owing to the very small number of excluded participants which would have an inconsequential effect on statistical inferences [44]. Participant characteristics are presented in **Table 2.** There were slightly more females than males, British citizens than non-British citizens, unemployed individuals than employed or retired individuals, and more individuals living in social- or private-rented accommodation than those in owner-occupied accommodation.

**Table 2:** Participant characteristics and differences in walking and MPA by socio-demographics (n = 5,923).

	Whole sample N(%)	Walk ≥5 days/week N(%)	MPA ≥5 days/week N(%)
**Sex**			
Male	2369 (40.0)	729 (30.8)	**495 (20.9)**
Female	3554 (60.0)	1013 (28.5)	**897 (25.2)**
**Age group**			
16–24	464 (7.8)	**191 (41.2)**	**141 (30.4)**
25–39	1650 (27.9)	**528 (32.0)**	**415 (25.2)**
40–54	1531 (25.8)	**482 (31.5)**	**399 (26.1)**
55–64	808 (13.6)	**239 (29.6)**	**178 (22.0)**
65+	1470 (24.8)	**302 (20.5)**	**259 (17.6)**
**Citizenship**			
British	5091 (86.0)	1512 (29.7)	**1276 (25.1)**
Non-British	832 (14.0)	230 (27.6)	**116 (13.9)**
**Employment**			
Working	1389 (23.5)	**535 (30.7)**	**485 (34.9)**
Not working	2773 (46.8)	**811 (29.2)**	**579 (20.9)**
Retired	1761 (29.7)	**396 (22.5)**	**328 (18.6)**
**Household**			
Adult	2364 (39.9)	**730 (30.9)**	**569 (24.1)**
Family	1885 (31.8)	**647 (34.3)**	**501 (26.6)**
Older	1674 (28.3)	**365 (21.8)**	**322 (19.2)**
**Tenure**			
Own	1379 (23.3)	**491 (35.6)**	**401 (29.1)**
Rent	4544 (76.7)	**1251 (27.5)**	**991 (21.8)**
**Vehicle ownership**			
Yes	1451 (24.5)	**492 (33.9)**	**459 (31.6)**
No	4472 (75.5)	**1250 (28.0)**	**933 (20.9)**

Bold typeface indicates significant difference at p<0.01 level controlling for area, distance to audit site and other demographic characteristics.

Only 29.4% of participants reported walking in the neighbourhood on at least 5 days/week and 23.5% reported participating in MPA on at least 5 days/week. Frequent walking was associated with participation in MPA (X^2^(1) = 955.49, p<0.001). Younger participants were more likely to report frequent walking and MPA, with a linear trend across the age groups. Participants in and out of employment were significantly more likely to walk or perform MPA on at least 5 days/week compared with retired participants and those with vehicles, were more likely to engage in frequent walking and MPA than others were. Those in family households were more likely to report frequent walking and MPA compared with those living in adult or older adult households, as were participants residing in owned accommodation compared with rented accommodation. Female participants were also significantly more likely to perform MPA on ≥5 days/week, as were British participants.

### Associations between social environmental factors and physical activity

**Table 3** presents findings for model 1 (containing main effects of environmental factors separately and adjusting for confounders) and model 2 (multivariate model containing main effects for all environmental factors, but no interaction terms, and adjusting for confounders) with walking as the outcome. Models were also conducted using continuous physical activity outcomes; associations were in the same direction and therefore are not reported here. In model 1, independent effects of all social factors were obtained, in the direction expected, i.e. stronger social factors associated with more walking. In model 2 (the multivariate model), three social factors retained significant positive associations with walking, they were: ‘social support’ (OR:1.22, 95%CI = 1.06–1.41, p<0.01), ‘social interaction’ (OR:1.25, 95%CI = 1.10–1.42, p<0.01) and ‘cohesion and safety’ (OR:1.78, 95%CI = 1.56–2.03, p<0.001). There was no effect of ‘trust and empowerment’ on walking in the multivariate model.

**Table 3:** Independent effects of social and physical environment factors on neighbourhood walking on at least 5 days/week (n = 5,923).

Environmental factor	% walking ≥5 days	Model 1			Model 2		
		OR	95% CI	p	OR	95% CI	p
**‘Social support’**							
Lower	25.4	1.00			1.00		
Higher	30.9	1.27	1.11–1.47	**.001**	1.22	1.06–1.41	**.006**
**‘Trust and empowerment’**							
Lower	27.8	1.00			1.00		
Higher	30.9	1.21	1.07–1.37	**.003**	1.10	0.97–1.25	.141
**‘Social interaction’**							
Weaker	25.0	1.00			1.00		
Stronger	32.8	1.34	1.18–1.52	**.000**	1.25	1.10–1.42	**.001**
**‘Cohesion and safety’**							
Lower	21.3	1.00			1.00		
Higher	35.6	1.89	1.66–2.15	**.000**	1.78	1.56–2.03	**.000**
**‘Aesthetics of built form’**							
Poorer	25.7	1.00			1.00		
Better	33.2	1.60	1.35–1.90	**.000**	1.47	1.22–1.77	**.000**
**‘Physical disorder’**							
More cues	26.4	1.00			1.00		
Fewer cues	32.1	1.43	1.20–1.70	**.000**	1.13	0.94–1.36	.190
**‘Aesthetics & maintenance of open space’**							
Poorer	28.4	1.00			1.00		
Better	29.9	1.42	1.22–1.66	**.000**	1.32	1.13–1.54	**.001**

Model 1: single social or physical environmental factor and covariates (sex, age, citizenship, employment status, tenure, mobility-limiting illness, vehicle ownership, distance to audit site and neighbourhood deprivation), adjusted for participant sub-area. Model 2: random intercept included in model to account for possible clustering within participant sub-area; model included all social and physical environmental factors and covariates.

**Table 4** presents findings for participation in MPA on ≥5 days/week. In model 1, there was a significant effect of ‘social interaction’ and ‘cohesion and safety’, only, both in the direction expected, i.e. stronger social factors associated with more physical activity. In model 2, there was an independent effect of three social factors: two were in the direction expected, namely ‘social interaction’ (OR:6.16, 95%CI = 5.14–7.37, p<0.001) and ‘cohesion and safety’ (OR:1.93, 95%CI = 1.65–2.27, p<0.001); one association was negative, namely ‘social support’ (OR:0.79, 95%CI = 0.67–0.94, p<0.01). There was no effect of ‘trust and empowerment’ in the multivariate model.

**Table 4:** Independent effects of social and physical environment factors on moderate physical activity on at least 5 days/week (n = 5,923).

Environmental factor	% MPA ≥5 days	Model 1			Model 2		
		OR	95% CI	p	OR	95% CI	p
**‘Social support’**							
Lower	22.8	1.00			1.00		
Higher	23.8	0.85	0.72–0.99	.034	0.79	0.67–0.94	**.007**
**‘Trust and empowerment’**							
Lower	20.5	1.00			1.00		
Higher	26.3	1.14	0.99–1.31	.063	1.14	0.98–1.33	.087
**‘Social interaction’**							
Weaker	7.4	1.00			1.00		
Stronger	35.6	6.68	5.59–7.97	**.000**	6.16	5.14–7.37	**.000**
**‘Cohesion and safety’**							
Lower	13.0	1.00			1.00		
Higher	31.5	2.38	2.04–2.77	**.000**	1.93	1.65–2.27	**.000**
**‘Aesthetics of built form’**							
Poorer	21.4	1.00			1.00		
Better	25.6	1.21	1.00–1.46	.050	1.02	0.82–1.27	.838
**‘Physical disorder’**							
More cues	19.2	1.00			1.00		
Fewer cues	27.3	1.94	1.60–2.36	**.000**	1.63	1.31–2.03	**.000**
**‘Aesthetics & maintenance of open space’**							
Poorer	25.4	1.00			1.00		
Better	22.5	1.32	1.11–1.56	**.001**	1.16	0.97–1.40	.107

Model 1: single social or physical environmental factor and covariates (sex, age, citizenship, employment status, tenure, mobility-limiting illness, vehicle ownership, distance to audit site and neighbourhood deprivation), adjusted for participant sub-area. Model 2: random intercept included in model to account for possible clustering within participant sub-area; model included all social and physical environmental factors and covariates.

### Associations between physical environmental factors and physical activity

**Table 3** presents results for associations between physical factors and walking for both model 1 and model 2. In model 1, all physical environment factors were related to walking in the direction expected, i.e. better conditions associated with more walking. In model 2 (multivariate model), only two physical environmental factors retained significance. ‘Aesthetics of the built form’ (OR:1.47, 95%CI = 1.22–1.77, p<0.001) and ‘aesthetics and maintenance of open space’ (OR:1.32, 95%CI = 1.13–1.54, p<0.01) had significant positive effects on regular walking. There was no independent effect of ‘physical disorder’.

**Table 4** presents results for MPA for model 1 and model 2. In model 1, ‘physical disorder’ and ‘aesthetics and maintenance of open space’ had positive effects on MPA. In model 2 (multivariate model), only ‘physical disorder’ was related to increased likelihood of participating in MPA on ≥5 days/week (OR:1.63, 95%CI = 1.31–2.03, p<0.001). There was no effect of ‘aesthetics of built form’ or ‘aesthetics and maintenance of open space’ on MPA in the multivariate model.

### Interactions between social and physical environment and impact on physical activity

Progressive removal of non-significant interaction terms in model 3 revealed significant interactive effects. There were two significant interactions between the social and built environments in relation to walking; between ‘trust and empowerment’ and ‘aesthetics and maintenance of open space’ (p<0.001); and between ‘cohesion and safety’ and ‘physical disorder’ (p<0.01).

For MPA, there were three significant interactions: between ‘trust and empowerment’ and ‘aesthetics and maintenance of open space’ (p<0.01) between ‘cohesion and safety’ and ‘physical disorder’ (p<0.01); and between ‘social interaction’ and ‘aesthetics of the built form’ (p<0.001). No other interaction terms between social and physical factors reached significance at p<0.01 for either walking or MPA.

In post hoc analyses (full results are presented in **S1 Table**), ‘cohesion and safety’ appeared to moderate the effect of ‘physical disorder’ on walking and MPA: ‘physical disorder’ only had a significant influence on activity outcomes when there was a higher level of ‘cohesion and safety’ (walking: OR = 1.50, 95%CI = 1.20–1.86, p<0.001; MPA: OR = 1.94, 95%CI = 1.53–2.47, p<0.001). In contrast, ‘cohesion and safety’ had a significant influence on activity outcomes regardless of ‘physical disorder’.

‘Social interaction’ moderated the influence of ‘aesthetics of built form’ on MPA: ‘aesthetics of built form’ only had an effect when ‘social interaction’ was high (OR = 1.40, 95%CI = 1.10–1.77, p<0.01) but ‘social interaction’ had a significant effect on MPA regardless of ‘aesthetics of built form’.

‘Trust and empowerment’ and ‘aesthetics and maintenance of open space’ appeared to operate synergistically upon walking and MPA: ‘aesthetics and maintenance of open space’ only had a significant influence on activity outcomes when ‘trust and empowerment’ was high (walking: OR = 2.12, 95%CI = 1.70–2.64, p<0.001; MPA: OR = 1.47, 95%CI = 1.15–1.87, p<0.01) and ‘trust and empowerment’ only had a significant effect when ‘aesthetics and maintenance of open space’ was high (walking: OR = 1.52, 95%CI = 1.30–1.77, p<0.001; MPA: OR = 1.29, 95%CI = 1.08–1.54, p<0.01).

## Discussion

In an adult population living in deprived communities in Glasgow, UK, independent and interactive effects of the social and physical environment on neighbourhood walking and participation in MPA were revealed. In models including a single environmental factor and adjusting for covariates and nesting in sub-area, all environment factors (‘social support’, ‘trust and empowerment’, ‘social interaction’, ‘cohesion and safety’, ‘aesthetics of built form’, ‘physical disorder’ and ‘aesthetics and maintenance of open space’) were significantly associated with increased likelihood of walking in the neighbourhood on ≥5 days/week. ‘Cohesion and safety’ had the largest effect, with participants reporting higher levels of cohesion being nearly twice as likely to regularly walk around their neighbourhood. In models with MPA as the outcome, ‘social interaction’, ‘cohesion and safety’, ‘physical disorder’ and ‘aesthetics and maintenance of open space’ were significant. ‘Social interaction’ had the largest independent effect on MPA, with participants reporting higher levels of ‘social interaction’ being more than 6 times more likely to meet national guidelines by participating in >30 minutes of MPA on ≥5 days/week. Interactive effects on walking and MPA were reported between i) ‘trust and empowerment’ and ‘aesthetics and maintenance of open space’ and ii) ‘cohesion and safety’ and ‘physical disorder’. There was also an interactive effect on MPA between ‘social interaction’ and ‘aesthetics or built form’.

Most independent effects were in the expected direction, with better quality social and physical environments related to an increased likelihood of frequent walking or MPA. The only negative relationship was between ‘social support’ and MPA which was non-significant in a univariate model (while also controlling for covariates) but attained significance in the multivariate model. This could be a spurious result (despite an alpha level p<0.01) or could be indicative of reverse causality whereby individuals with a greater need for social support (and therefore higher self-reported levels) are less able or likely to participate in higher-intensity physical activity. Results replicate previous findings from the GoWell sample where individual measures of safety after dark, informal social control (intervening in harassment), belonging to the neighbourhood and cohesion between residents of different backgrounds had significant positive independent effects on neighbourhood walking [45]. Findings also support those from other populations. For example, Shelton et al. found in a sample of 1,112 adults in low-income communities in the USA that participants who reported stronger social networks (and thereby higher levels of social interaction) also performed higher levels of pedometer-assessed physical activity [46]. Positive associations between objectively-measured aesthetics of the built form and open space and self-reported walking reflect previous findings from the IPEN study across 17 cities in 12 countries which found an effect of self-reported perceived aesthetics and self-reported walking for transport for ≥150 minutes per week [47]. As noted in Kerr et al., an effect of micro-scale features of the physical environment such as aesthetics presents a potentially low-cost intervention strategy [47].

There were some notable differences between independent environmental effects on neighbourhood-based walking and MPA. In models including all environmental factors and covariates, there was an effect of all social environmental factors except ‘trust and empowerment’ on both activity outcomes. However, effects on MPA, compared with neighbourhood-based walking, were much stronger for ‘social interaction’, slighter stronger for ‘cohesion and safety’ and in the opposite direction for ‘social support’. This is somewhat surprising as it might be expected that if there are neighbourhood effects on activity, the local neighbourhood environment would be more closely associated with activity performed in the neighbourhood. However, it is likely that although the measure of MPA was not context-specific, the majority of activity reported by participants was in fact performed in the neighbourhood and encompassed walking and additional activities such as household chores, gardening or using local physical activity facilities [48]. There were also differences in the effect of the built environment on activity outcomes:–‘physical disorder’ only had an independent effect on MPA while ‘aesthetics of built form’ and ‘aesthetics and maintenance of open space’ only had an effect on walking. It is possible these differences can be attributed to the activities included in MPA other than walking, such as use of physical activity facilities, and the comparative importance of a pleasant and attractive public realm for walking-based activities. Differences might be explained by the extent to which the physical environment interacted with the social environment, as discussed below.

Interactions between i) ‘trust and empowerment’ and ‘aesthetics and maintenance of open space’ and ii) ‘cohesion and safety’ and ‘physical disorder’ were found for both walking and MPA. An interactive effect of ‘social interaction’ and ‘aesthetics of built form’ was also reported for MPA. Post hoc analyses suggested that the effect of ‘physical disorder’ appeared to operate through ‘cohesion and safety’, with fewer cues of disorder supporting higher levels of activity only when participants viewed their neighbourhoods as socially-cohesive and safe. Post-hoc tests showed that the largest effect was observed for participants reporting high levels of ‘cohesion and safety’ and living in areas with fewer cues of ‘physical disorder’. These participants were 2.2 times more likely to walk or perform MPA on at least 5 days/week than participants living in areas with fewer cues of ‘physical disorder’ but reporting lower levels of ‘cohesion and safety’. There was no independent effect of ‘physical disorder’.

The effect of ‘aesthetics of built form’ on MPA was similarly constrained by ‘social interaction’: only when participants had higher levels of social interaction did the positive influence of aesthetics of the built form occur–there was no effect of ‘aesthetics of built form’ when ‘social interaction’ levels were lower. The largest effect was apparent when both ‘social interaction’ and ‘aesthetics of built form’ were high, in this case, participants were 10 times more likely to perform MPA on 5 days/week, than participants reporting low levels of ‘social interaction‘ in areas with better ‘aesthetics of built form’.

Previous research has documented an association between physical disorder and anti-social behaviour [49,50], speculating that cues of neglect and ‘physical incivilities’ suggest residents do not abide by social norms and are therefore more likely to engage in anti-social or criminal behaviour [29]. It is therefore feasible that physical disorder acts as a cue to harmonious relationships between neighbours, or that harmonious relationships encourage individuals to take care of their physical environment. Findings from this study suggests that when individuals perceived low levels of safety and cohesion there was no effect of physical disorder, possibly because cues for safety and cohesion that were usually elicited from physical disorder were overridden. However, physical disorder had an effect on physical activity in individuals reporting high levels of safety and cohesion; possibly disorder was neutralised as a cue for safety but still created an uninviting, smelly or dirty environment.

Additionally, it could be speculated from results that aesthetically-pleasing built form only supported MPA when individuals had many friends and neighbours because visually interesting and attractive environments and buildings were only important when they could be used for recreational team sports or joint activities (e.g. an attractive green space or leisure centre). Because assessments of the built form were objectively reported by auditors, it suggests that an interactive effect is not due to individuals with higher levels of social interaction feeling more attached to the neighbourhood and therefore perceiving the local physical environment in a more positive light. However, it is possible that an attractive built form might be more readily felt by individuals with higher levels of social interaction, who then are more motivated to use, and be active in, their local environment.

These interactive effects support a key tenet of socioecological models of physical activity and are substantiated by emerging evidence from the USA. For example, perceived safety appeared to interact with walkability, mitigating the effect of neighbourhood walkability whereby walkability exerted a smaller effect on MVPA when levels of perceived safety were held constant (e.g. constantly high) than did the effect of safety when levels of walkability were held constant [28]. Such findings suggest that aspects of the social environment may be more important than physical aspects in encouraging individuals to be active within a deprived context. Intervening to increase physical activity through reduction of physical disorder or creation of more attractive and interesting built form might therefore be successful only when the social environment is also supportive of physical activity.

In the current study, ‘trust and empowerment’ and ‘aesthetics and maintenance of open space’ appeared to act synergistically upon walking and MPA: ‘aesthetics and maintenance of open space’ was only important when there was a high level of ‘trust and empowerment’ and vice versa. It is feasible that these aspects of the social and physical environment reinforce one another. For example, residents who trust each other and feel empowered may have the capability and motivation to maintain and/or advocate for attractive open space, resulting in attractive open spaces which prompt further mutual trust and empowerment. Similarly, in Kaczynski and Glover’s study of 380 adults in Canada, the highest levels of walking for transport or recreation were reported in neighbourhoods with high walkability (a composite measure including macro- and micro-scale physical features) *and* high levels of cohesion and trust [51]. To our knowledge, this is the first time an interactive effect of community trust and empowerment and quality of open space on physical activity has been reported in a deprived setting in the UK.

Findings should be replicated and further explored within deprived contexts and using longitudinal or quasi-experimental study designs in order to establish the reliability of associations and unpick the direction of causality. If supported by future research, interactive environmental effects on physical activity may have important implications for future research and policy within the field of active design. Firstly, if unmeasured factors moderate or act synergistically upon measured factors, variance arising from the influence of unmeasured factors might underpin some of the reported inconsistency within the physical activity literature examining neighbourhood effects. This is highlighted by the independent effects reported in the multivariate models that did not include interactive effects. Insight into these relationships can contribute to the development of theoretical frameworks of neighbourhood influence on physical activity.

Secondly, as discussed, it is possible that simultaneously targeting aspects of social and physical environment that work together to influence activity will harness the largest effects on walking and MPA. For example, interventions may be most successful when they simultaneously target aspects of the environment or deploy specific strategies in neighbourhoods with existing social/physical environmental characteristics conducive to activity. Findings are therefore supportive of a holistic approach to regeneration which includes strategies for both social and physical regeneration or leverages the effect of existing supportive social contexts.

### Strengths and limitations

It is important that interventions or policy employing active design principles are informed by timely research that is, as far as possible, specific to resident populations and contexts. However, context-specific research also limits generalizability and it is necessary to bear in mind that assessments of the environment in this study were relative: a high level of trust and empowerment or better aesthetics of built form may not be regarded as ‘high quality’ in another context. Moreover, because analyses were cross-sectional, it is not possible to assess the causal direction of relationships: better quality social and physical environments might be an outcome of increased physical activity or a third factor might act upon both the environment and physical activity.

Neighbourhood deprivation, participant employment status and vehicle ownership were controlled for in statistical models as proxy measures of socio-economic status but we were unable to control for individual income or deprivation due to a lack of complete data (<25% of sample). Individual income could act on the perceived social environment and physical activity behaviour but is unlikely to act on the physical environment beyond any effect of neighbourhood deprivation, unless there is spatial clustering of income or individual deprivation within neighbourhoods. Longitudinal analyses and natural experiments will provide insight into the causal pathways between the environment and physical activity although it is conceivable that the relationship between the environment and physical activity is not linear, but dynamic and complex, with environmental aspects being both determinants and outcomes of physical activity in the neighbourhood.

A large sample increases the reliability and generalisability of the results and is necessary to test for an interaction effect [18,43]. Obtaining a large sample from deprived neighbourhoods is therefore a major strength of this study. However, it is expensive and often unfeasible to collect objective measurements of physical activity in large samples. Therefore, self-reported physical activity was used in this study, potentially reducing the validity and reliability of this measure. Nonetheless, previous studies using single-item self-report physical activity measures have shown that they can have adequate criterion validity against accelerometry and moderate validity and strong repeatability against more extensive self-report tools [52,53]. In addition, there is a valuable congruence in using self-reported data to assess adherence to physical activity guidelines (e.g. participation in physical activity on approximately 5 days/week) which were themselves developed using self-reported physical activity data as a basis to estimate associated health benefits [54,55]. Examining environmental influences on two physical activity outcomes adds strength to our findings, which corroborated one another in terms of strength and direction of effects. Neighbourhood was generically defined in the survey as the area within a 5–10 minutes’ walk of the respondent’s home. However, it should be acknowledged that in comparison to the non-specific MPA outcome, the context-specific physical activity outcome (neighbourhood-based walking) might be vulnerable to response bias in that individual factors or physical activity levels could influence the exact size and shape of participants’ own self-defined interpretation of their neighbourhood. Future research should aim to replicate these results using objective measures of physical activity and explore whether the effect differs for various domains of activity (e.g. transport, recreational).

Structural elements of the physical environment that are typically used to assess walkability (e.g. density, connectivity and land use) were not within the scope of the current study. Instead, this study responded to recent evidence that quality-related features of the physical environment (e.g. aesthetics, maintenance, disorder) may present more affordable and practical targets for neighbourhood interventions to increase physical activity and alleviate health inequalities by lessening reported disparities in these features between lower- and higher-income communities [26,27]. Nonetheless, results should be interpreted in light of possible additional influence of structural macro-scale elements of the environment on neighbourhood walking and MPA.

## Conclusions

Findings reveal independent and interactive associations between the quality of the social and physical environment and neighbourhood walking and MPA. Results demonstrate the importance of simultaneous consideration of multiple aspects of the social and physical environment in both research and active design policy. To our knowledge, this is the first examination of interactive effects of the social and physical environment in a sample of adults living in income-deprived neighbourhoods in the UK.

## Supporting information

S1 Fig GoWell neighbourhood locations in 2006. Background map sourced from Google Maps; www.google.co.uk/maps. Accessed February 2017.
(DOCX)

S1 Table Results for post-hoc tests examining effect of selected environment factor on walking and moderate physical activity. Models were conducted by stratified group and included selected environment factors and covariates (sex, age, citizenship, employment status, tenure, mobility-limiting illness, vehicle ownership, distance to audit site and neighbourhood deprivation), adjusted for participant sub-area.
(DOCX)
