# Supplementary material for: Cross-sectional interactions between quality of the physical and social environment and self-reported physical activity in adults living in income-deprived communities
Source: PLoS One. 2017 Dec 14;12(12):e0188962. doi: 10.1371/journal.pone.0188962 (PMC5730220; doi:10.1371/journal.pone.0188962)
Supplement: S1 Fig — Background map sourced from Google Maps; www.google.co.uk/maps. Accessed February 2017. (DOCX) [file pone.0188962.s001.docx]

S1 Fig: GoWell Neighbourhood locations in 2006


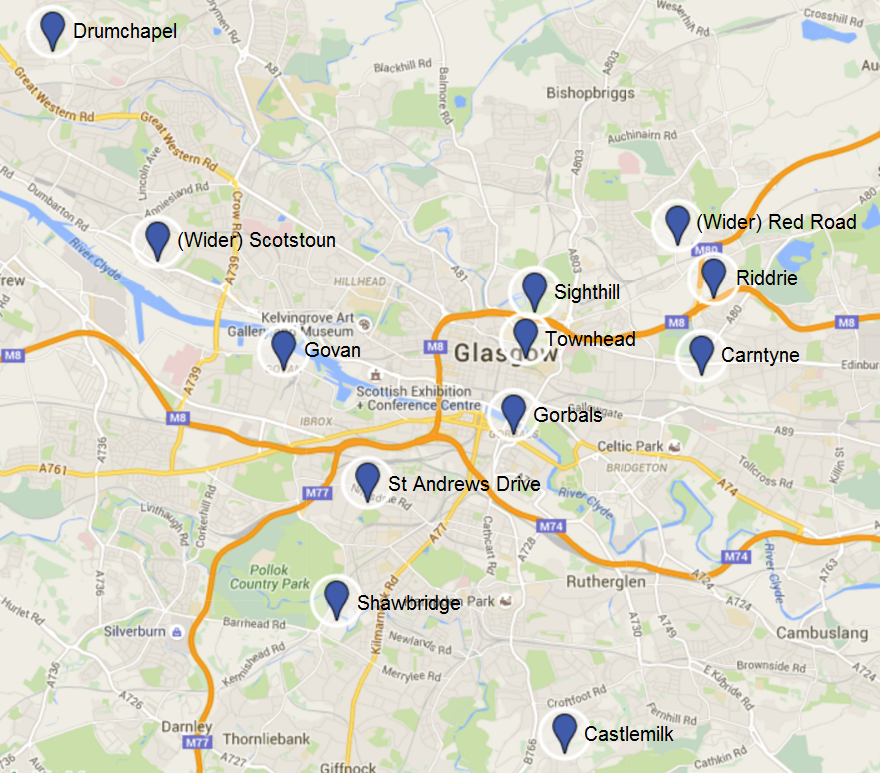


Background map sourced from Google Maps; www.google.co.uk/maps. Accessed February 2017.
