# Supplementary material for: Cross-sectional interactions between quality of the physical and social environment and self-reported physical activity in adults living in income-deprived communities
Source: PLoS One. 2017 Dec 14;12(12):e0188962. doi: 10.1371/journal.pone.0188962 (PMC5730220; doi:10.1371/journal.pone.0188962)
Supplement: S1 Table — Models were conducted by stratified group and included selected environment factors and covariates (sex, age, citizenship, employment status, tenure, mobility-limiting illness, vehicle ownership, distance to audit site and neighbourhood deprivation), adjusted for participant sub-area. (DOCX) [file pone.0188962.s002.docx]

**S1 Table: Results for post-hoc tests examining effect of selected environment factor on walking and moderate physical activity**

| Stratification | N | Exposure factor | Walking | | | MPA | | |
| --- | --- | --- | --- | --- | --- | --- | --- | --- |
|  |  |  | OR | 95% CI | p | OR | 95% CI | p |
| ‘Trust and empowerment’  Lower | 2,830 | ‘Aesthetics & maintenance of open space’  Lower  Higher | 1.00  0.84 | 0.68 – 1.04 | .109 | 1.00  1.00 | 0.78 – 1.27 | .984 |
| ‘Trust and empowerment’  Higher | 3,093 | ‘Aesthetics & maintenance of open space’  Lower  Higher | 1.00  2.12 | 1.70 – 2.64 | **.000** | 1.00  1.47 | 1.15 – 1.87 | **.002** |
| ‘Aesthetics & maintenance of open space’  Poorer | 1,995 | ‘Trust and empowerment’  Lower  Higher | 1.00  0.76 | 0.62 – 0.94 | .012 | 1.00  0.88 | 0.68 – 1.13 | .311 |
| ‘Aesthetics & maintenance of open space’  Better | 3,928 | ‘Trust and empowerment’  Lower  Higher | 1.00  1.52 | 1.30 – 1.77 | **.000** | 1.00  1.29 | 1.08 – 1.54 | **.004** |
| ‘Cohesion and safety’  Lower | 2,558 | ‘Physical disorder’  More  Fewer | 1.00  1.10 | 0.85 – 1.41 | .484 | 1.00  1.10 | 0.85 – 1.41 | .484 |
| ‘Cohesion and safety’  Higher | 3,365 | ‘Physical disorder’  More  Fewer | 1.00  1.50 | 1.20 – 1.86 | **.000** | 1.00  1.50 | 1.20 – 1.86 | **.000** |
| ‘Physical disorder’  More cues | 2,778 | ‘Cohesion and safety’  Lower  Higher | 1.00  1.58 | 1.31 – 1.91 | **.000** | 1.00  1.58 | 1.31 – 1.91 | **.000** |
| ‘Physical disorder’  Fewer cues | 3,145 | ‘Cohesion and safety’  Lower  Higher | 1.00  2.20 | 1.83 – 2.65 | **.000** | 1.00  2.20 | 1.83 – 2.65 | **.000** |
| ‘Social interaction’  Lower | 2,537 | ‘Aesthetics of built form’  Lower  Higher | - | - | **-** | 1.00  0.59 | 0.39 – 0.91 | .016 |
| ‘Social interaction’  Higher | 3,386 | ‘Aesthetics of built form’  Lower  Higher | - | - | **-** | 1.00  1.40 | 1.10 – 1.77 | **.005** |
| ‘Aesthetics of built form’  Poorer | 3,017 | ‘Social interaction’  Lower  Higher | - | - | **-** | 1.00  4.93 | 3.93 – 6.18 | **.000** |
| ‘Aesthetics of built form’  Better | 2,906 | ‘Social interaction’  Lower  Higher | - | - | **-** | 1.00  10.67 | 7.88 – 14.46 | **.000** |

Models were conducted by stratified group and included selected environment factors and covariates (sex, age, citizenship, employment status, tenure, mobility-limiting illness, vehicle ownership, distance to audit site and neighbourhood deprivation), adjusted for participant sub-area.
